# Detection of aberrant methylation of a six-gene panel in serum DNA for diagnosis of breast cancer

**DOI:** 10.18632/oncotarget.7608

**Published:** 2016-02-23

**Authors:** Ming Shan, Huizi Yin, Junnan Li, Xiaobo Li, Dong Wang, Yonghui Su, Ming Niu, Zhenbin Zhong, Ji Wang, Xianyu Zhang, Wenli Kang, Da Pang

**Affiliations:** ^1^ Department of Breast Cancer Surgery, the Affiliated Tumor Hospital of Harbin Medical University, Harbin, P.R. China; ^2^ Translational Medicine Research and Cooperation Center of Northern China, Heilongjiang Academy of Medical Sciences, Harbin, P.R. China; ^3^ Department of Epidemiology and Biostatistics, Harbin Medical University, Harbin, P.R. China; ^4^ Department of Pathology, Harbin Medical University, Harbin, P.R. China; ^5^ College of Bioinformatics Science and Technology, Harbin Medical University, Harbin, P.R. China; ^6^ Department of Oncology, General Hospital of Heilongjiang Province Land Reclamation Headquarters, Harbin, P.R. China

**Keywords:** breast cancer, DNA methylation, diagnosis, MethyLight

## Abstract

Detection of breast cancer at an early stage is the key for successful treatment and improvement of outcome. However the limitations of mammography are well recognized, especially for those women with premenopausal breast cancer. Novel approaches to breast cancer screening are necessary, especially in the developing world where mammography is not feasible. In this study, we examined the promoter methylation of six genes (SFN, P16, hMLH1, HOXD13, PCDHGB7 and RASSF1a) in circulating free DNA (cfDNA) extracted from serum. We used a high-throughput DNA methylation assay (MethyLight) to examine serum from 749 cases including breast cancer patients, patients with benign breast diseases and healthy women. The six-gene methylation panel test achieved 79.6% and 82.4% sensitivity with a specificity of 72.4% and 78.1% in diagnosis of breast cancer when compared with healthy and benign disease controls, respectively. Moreover, the methylation panel positive group showed significant differences in the following independent variables: (*a*) involvement of family history of tumors; (*b*) a low proliferative index, ki-67; (*c*) high ratios in luminal subtypes. Additionally the panel also complemented some breast cancer cases which were neglected by mammography or ultrasound. These data suggest that epigenetic markers in serum have potential for diagnosis of breast cancer.

## INTRODUCTION

Breast cancer is a complex and heterogeneous disease and one of the leading causes of death among women. Detection of breast cancer at an early stage is the key for successful treatment and improvement of outcome. Although a significant decline in breast cancer mortality between 1992 and 1996 is believed to be due, in part, to early diagnosis by screening mammography, however the limitations of mammography [1] are well recognized, especially for those women with premenopausal breast cancer. In developing countries, the extensive laboratory and clinical infrastructure required for mammographic screening, as well as the high cost of mammography precludes such an approach. Further, given that the majority of women in many developing countries are under the age of 40 years, the problem of detecting premenopausal breast cancer is of particular important in such settings. Despite the availability of mammography and the prevalence of self-examination, additional benefits can still be gained from additional screening methodologies [2]. Therefore, developing novel approaches for the early diagnosis of breast cancer has important clinical implications.

Epigenetic change, including DNA methylation, is one of the most common molecular alterations in human neoplasia, including breast cancer [3]. CpG islands located in promoter regions of tumor suppressive genes are generally unmethylated in normal cells. However, in cancer cells, aberrant hypermethylation of these promoter regions is associated with transcriptional silencing. Hypermethylation is therefore an alternative mechanism for the inactivation of tumor suppressor genes [4, 5]. Since gene hypermethylation has been found to be a common and early event in many tumor types, including breast tumors [6, 7], it has emerged as a promising target for detection strategies involving clinical specimens [8, 9]. Serum or plasma is a more readily accessible bodily fluid, and provision of a specimen does not require the presence of a specialist. DNA is known to be released into serum/plasma, which is enriched for tumor DNA in cancer patients [10]. Several recent studies have shown that it is possible to detect tumor-specific methylation alterations in serum DNA from head and neck, lung, and colon cancer patients. Importantly, tumor cell-specific DNA methylation in serum is not limited to patients with metastatic cancer but is also present in serum from patients with early organ-confined tumors [11–14]. Neoplastic DNA in serum most likely arises from cells that have left the site of the primary lesion and have invaded the circulatory system but lack the capacity of metastasis to new organs, or it may be released from the primary tumor as free DNA from nonviable (apoptotic) neoplastic cells [8, 9].

However, in spite of the promise of such biomarkers, there are several barriers that impede fast progress toward their clinical application. Major limitations to further development for clinical application might be that these studies investigated only small numbers of methylated targets, or fewer breast cancer and matched control specimens, and validation with larger patient cohorts was not pursued [9, 15]. Furthermore, many studies have focused on investigating the methylation patterns in serum from healthy women and those with breast cancer, but only rare studies using benign breast diseases as control to identify the potential clinical applications of serum DNA methylation. Other limitations include the utilization of different technologies by different laboratories with a range of detection sensitivity, varying emphasis on quantitation, and the utilization of different sample processing methodologies and different reference materials as controls for the analysis of hypermethylation degree by the same technology [16]. In our paper unpublished, we had chosen lots of genes hypermethylated in breast cancer tissue and hypomethylated in the match normal breast tissue from the TCGA database. However many of them didn't show hypermethylation in our breast cancer patients. The genes we selected, SFN, P16, hMLH1, PCDHGB7 and RASSF1a had the most frequency of methylation in breast cancer samples in our previous research and the methylation of some of the genes happened at the early stage of breast cancer. Additionally, they have previously been shown to undergo cancer-specific methylation according to reports of clinical or fundamental studies [17–22]. So in this study, the promoter methylation of the six-gene panel was examined by using serum collected from more than 749 cases, including breast cancer patients, patients with benign breast diseases and healthy women to prove if they are suitable for diagnostic biomarkers of breast cancer in serum. Moreover we added a new methylated site of HOXD13 in the promoter region, which didn't exist in our previous research, but showed significant methylation in serum from breast cancer patients comparing to benign breast diseases patients and healthy women in this study (P<0.05). We also compared our gene panel diagnosis alongside with mammography and ultrasound, which were routine diagnostic tools for breast cancer in clinic. Our study suggests that epigenetic markers in serum provide reliable targets for breast cancer diagnosis and optimized epigenetic biomarkers would have great potential in clinical application.

## RESULTS

**Figure 1:**
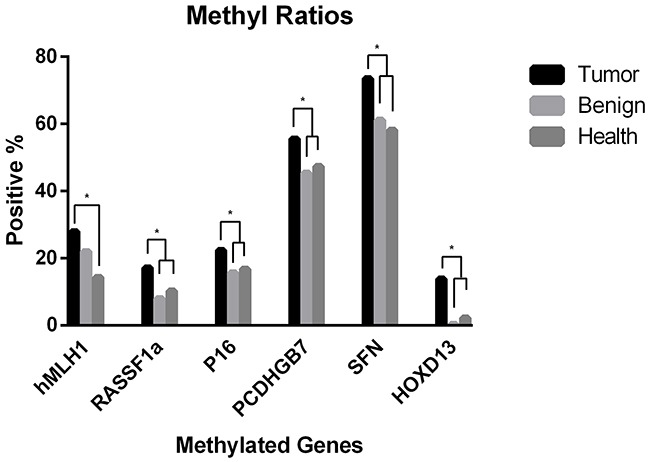
Methylation frequency of all candidate markers in serum from breast cancer versus non-breast tumors * *P* <0.05.

In this study, we assessed the promoter methylation of an six-gene panel (SFN, P16, hMLH1, HOXD13, PCDHGB7 and RASSF1a) in serum samples by using MethyLight, to investigate whether it could be used for diagnosis of breast cancer or not. All of these genes are representatives of a variety of cellular pathways that are involved in cancer, including DNA binding, cell cycle/checkpoint control, developmental regulation, chromatin binding and cytokine activity (Supplementary Table S1). An overview of the methylation frequency in the studied serum samples of all the three groups is given in Table 1. HOXD13 is rarely methylated in the sera from breast cancer patient group (13.81%). However, the unmethylation frequency of HOXD13 is significantly increased in the sera from healthy women (97.55%) and patient group with benign breast diseases (99.58%). Conversely PCDHGB7 and SFN exhibited better sensitivities in breast cancer (55.60% and 73.51%), but the specificity was unsatisfied. Most of the candidate markers were significantly methylated in sera from breast cancers than non-breast tumors (P<0.05, Figure 1).

**Table 1:** Sensitivity and specificity of serum-based detection of aberrantly methylated genes in all groups

*Genes*	*Sensitivity in Breast Cancer*		*Specificity in Benign Breast Diseases*		*Specificity in Healthy Controls*	
	*Methylation Positive*	*%*	*Methylation Negative*	*%*	*Methylation Negative*	*%*
***hMLH1***	75 of 268	*27.99*	184 of 236	*77.97*	210 of 245	*85.71*
***RASSF1a***	46 of 268	*17.16*	217 of 236	*91.95*	220 of 245	*89.67*
***P16***	60 of 268	*22.39*	199 of 236	*84.32*	204 of 245	*83.27*
***PCDHGB7***	149 of 268	*55.60*	129 of 236	*54.66*	129 of 245	*52.65*
***SFN***	197 of 268	*73.51*	92 of 236	*38.98*	102 of 245	*41.63*
***HOXD13***	37 of 268	*13.81*	235 of 236	*99.58*	239 of 245	*97.55*

According to the previous researches [23], multiple marker combinations were able to improve the sensitivity and specificity of methylation biomarkers for tumor diagnosis. So next we performed ROC curve analysis for all the samples to determine the sensitivity and specificity of the six-gene ensemble.

**Figure 2:**
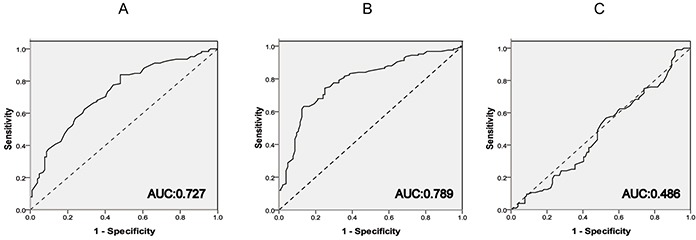
ROC curve analysis of the six-gene panel among all the groups **A.** Between Breast Cancer and Health; **B.** Between Breast Cancer and Benign Breast Diseases. **C.** Between Benign Breast Diseases and Health.

The R RandomForest analysis randomly divided all the cases into two data set, including the training set (128 breast cancer cases *vs* 114 benign breast disease cases and *vs* 112 healthy women cases) and the test set (125 cancer *vs* 104 benign disease and *vs* 104 health). The optimal threshold value for the six-target union to distinguish cancer from controls (benign diseases and health) was determined firstly in the training data set. Then the analysis model was validated in the test data set to calculate the AUC value, the sensitivity and specificity. There were no significant differences between the training and test data set with respect to any of the measured demographic or clinicopathologic characteristics or with respect to tumor stage among the cases. According to this analysis, the six-marker panel could discriminate between breast cancer patients and healthy women with a sensitivity of 79.6% and a specificity of 72.4% (AUC, 0.727 (95% CI, 0.712 to 0.742), P<0.001, Figure 2A). Additionally, the six-marker panel is able to distinguish breast cancer patients from women with benign breast diseases with higher specificity and sensitivity (78.1% and 82.4%; AUC, 0.789 (95% CI, 0.775 to 0.797), P<0.001, Figure 2B). It is worth noting that there was no significant differences between benign breast diseases and healthy women when using this panel (AUC, 0.486 (95% CI, 0.471 to 0.492), P>0.05, Figure 2C). Different AUC values of these six gene markers were also tested and the six-gene panel remains the top.

**Figure 3:**
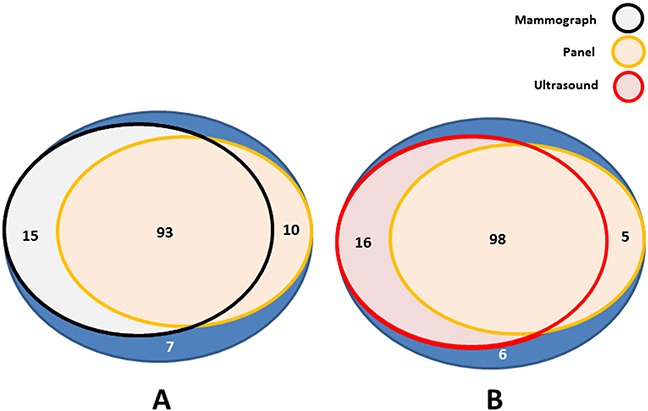
Positive diagnosis of breast cancer by mammography, ultrasound and six-gene panel in breast cancer group **A.** The diagnosis consistency of breast cancer between panel methylation and mammography. **B.** The diagnosis consistency of breast cancer between panel methylation and ultrasound.

The methylation status of the six-gene panel in the breast cancer patients of the test set was further analyzed for association with known clinicopathologic characteristics of breast cancer, including age at diagnosis, family history of tumors, tumor grade, tumor size, node status, and hormone-receptor status, etc (Table 2). The family history of tumors means the first degree relatives of patients have or died of malignant tumors, including lung cancer, hepatocarcinoma, breast cancer and so on. There are more patients with low ki-67 index in the methylation panel positive group. And the percentage of ki-67 positive staining is lower than 30% in most of them. So statistical analysis revealed significant differences in the following independent variables: (*a*) involvement of family history of tumors (P= 0.0249); (*b*) a low proliferative index, ki-67 (P= 0.0356); (*c*) high methylated frequency in luminal subtypes (P= 0.0319). In the methylation panel positive group, it occupied 59.22% in luminal-A subtype and 25.24% in luminal-B subtype. However in the methylation panel negative group, it became lower and showed 36.36% in luminal-A type and 22.73% in luminal-B type. The rest of the variables analyzed displayed no statistically significant differences. Next we investigated the consistency among the six-gene methylation panel, mammography, and ultrasound for breast cancer diagnosis by Kappa conformance test. Comparing mammography with ultrasound, significant consistency was observed (P=0.0012), and the consistency rate was high (0.856, shown in Table 3). In further calculation of the consistency between six-gene panel and either mammography or ultrasound, there was also significant statistical consistency. Moreover, in the mammography diagnosis group, there were 10 breast cancer cases that were panel methylation positive but with mammography diagnosis results negative for breast cancer. In the ultrasound group, 5 breast cancer cases were neglected. However, the panel methylation test method could show that these people had breast cancer (Figure 3). In addition, we further divided tumor sizes into two groups, decreased from >2 cm to ≤1 cm, the sensitivity of mammography declined from 88.24% to 81.82% (Table 4). On the other hand, the diagnostic sensitivity of methylated gene panel increased modestly along with tumor size decrease.

**Table 2:** Pathological characteristics of breast carcinomas showing a statistically significant difference between the six-marker panel positive and negative groups

Factors	Panel Positive	%	Panel Negative	%	*P Value*
**Patient characteristics**					
* Age at diagnosis*	*50.86±11.30*		*49.44±8.66*		*0.3549*
* BMI at diagnosis (kg/m^2)*	24.06±3.97		23.10±3.08		*0.0788*
**Frequencies**					**0.0249**
* Family History of Tumor*	29	28.16	1	4.55	
* no Family History of Tumor*	74	71.84	21	95.45	
**Tumor characteristics**					
* Size*					*0.6313*
* ≤2.0*	39	37.86	10	45.45	
* >2.0*	64	62.14	12	54.55	
* Grade*					*0.9533*
* Well differentiated*	5	4.86	1	4.55	
* Moderately differentiated*	82	79.61	17	77.27	
* Poorly differentiated*	16	15.53	4	18.18	
* Lymphatic-metastasis*					*0.562*
* No*	48	46.6	13	59.09	
* ≤3*	26	25.24	4	18.18	
* >3*	29	28.16	5	22.73	
* Ki-67*					**0.0356**
* ≤30%*	93	90.29	16	72.73	
* >30%*	10	9.71	6	27.27	
* P53*					*0.4211*
* Positive*	24	23.3	7	31.81	
* Negative*	79	76.7	15	68.19	
* Subtypes*					**0.0319**
* Luminal-A*	61	59.22	8	36.36	
* Luminal-B*	26	25.24	5	22.73	
* Her-2*	10	9.71	4	18.18	
* Triple-negative*	6	5.83	5	22.73	

**Table 3:** Consistency tests among panel, mammography and ultrasound groups

Group	Consistency Rate	*P value*
**Mammography and Ultrasound**	0.856	***0.0012***
**Panel and Mammography**	0.8	***0.006***
**Panel and Ultrasound**	0.832	***0.0008***

**Table 4:** Diagnostic sensitivities for mammography and six-gene methylation panel in different tumor sizes

Diagnosis Method	Tumor Size	Sensitivity (%)
***Mammography***		
	>2cm	60/68 (88.24)
	≤1cm	9/11 (81.82)
***Six-gene Methylation Panel***		
	>2cm	54/67 (80.60)
	≤1cm	12/14 (85.71)

## DISCUSSION

To investigate new methods for early breast cancer diagnosis, we tested an six-gene panel by using a blood-based PCR assay for methylated circulating free DNA (cfDNA) in two independent serum sets with a total of 749 serum samples, including sera collected from breast cancer patients, women with benign breast diseases and healthy women. Among these six genes, four of them (SFN, hMLH1, HOXD13 and PCDHGB7) have not been investigated previously as blood-based biomarkers for breast cancer diagnosis. However, the high methylation frequency of these genes in breast tumor tissues, including DCIS, found in our previous research implied their potential application for early breast cancer detection. Circulating serum DNA, presumably shed from the original primary tumor, can be retrieved and tested for genetic and epigenetic alterations. Previous studies have reported various genetic and epigenetic alterations in matched samples from tumor tissue and serum in patients with cancer [24–26]. Although knowledge of the underlying mechanism of this circulating DNA is still limited [27], some evidences suggested that the cfDNA is released from the tumor as a glyconucleoprotein complex that may protect it from degradation by nucleases [28]. It remains unclear whether release of tumor DNA into serum is associated with tumor necrosis, apoptotic cell death, or other selective cellular processes.

Interestingly, in this study, aberrant methylation was detected not only in patients with breast cancer but also in a small proportion of control subjects. Methylation of several genes has been reported previously in nonmalignant tissues and serum DNA of smokers. The presence of aberrant methylation in serum DNA could be a marker of disease (an early neoplastic effect), exposure (a biologic effect of an environmental factor) or both. The presence of aberrant methylation in healthy subjects may reflect chronic exposure to still unidentified environmental carcinogenic factors or inherited oncogenic mutations, because we found more breast cancer patients who had family history of tumors in the methylation positive panel group (28.13%, *see in* Table 2). Longitudinal epidemiologic studies confirming the association between the risk of developing precancerous lesions and the aberrant gene methylation and also demonstrated that the basis of methylation was related to environmental factors and oncogenetic (such as BRCA1/BRCA2) mutations. Thus, detection of methylation may help to identify high-risk individuals so that the breast cancer could be early diagnosed by using more intensive standard evaluation methods. Moreover, cfDNA could originate from other normal organs in the body and it is also released during cell aging, apoptosis or other procedure. As previous researches reported [18, 29], different organs showed tissue-specific methylation patterns. In this study, we identified low frequency of genes methylation in the serum from control groups, which may release from other tissues than the breast tissue. But it is obviously that the frequency of the gene methylation from the breast cancer patients was significantly higher than that in control groups. At last, these data provide strong, albeit indirect, evidence that the DNA containing the methylation of the specified gene originates from the primary breast cancer and is not affected by the serum DNA from nonmalignant tissues. Accumulating data on these methylation markers including the six genes tested in this study (HOXD13, SFN, RASSF1a, P16, PCDHGB7, and hMLH1) is of interest for further evaluation as serum- or serum-based biomarkers for the detection and monitoring of breast cancer patients.

Detection of methylation in circulating DNA depends on the ability of the assay to detect methylated DNA in a background of wild-type DNA (estimated at >1:1000). In our experience and that reported by others [30, 31], MethyLight is more sensitive than conventional MSP. Methylation changes in carcinogenesis are often heterogeneous, and no single gene has been found methylated in every breast cancer specimen so far. Furthermore, in most studies investigating methylation levels by using single gene, the sensitivity has been generally low [17]. Therefore, it is considered that a panel of genes for breast cancer screening procedures would improve the sensitivity. In our current study, we were able to define a biomarker panel with the significant values for breast cancer diagnosis by investigation of a strong and realistic cohort of control samples (including patients with benign breast diseases and healthy women). Our six-gene panel indicated significantly high sensitivity of 82.4% and also high specificity of 78.1% with an AUC of 0.789. Meanwhile according to the negative result of the ROC analysis between benign breast diseases and healthy women, this panel showed better clinical application for breast cancer diagnosis.

Regarding mammography, with a sensitivity of >70% and a specificity of >85%, a reliable biomarker panel for early detection of breast cancer in cfDNA need to reach comparable values. In our study, the mammography used for breast cancer diagnosis achieved the sensitivity of 86.4%. The sensitivity of ultrasound was a little higher than mammography, since ultrasound for breast cancer diagnosis may be suitable for dense breast tissue and small sized breast tumors [32, 33]. However, the accuracy of ultrasound diagnosis depends on the experience of radiologists, so it is not objective and will be limited in the clinical application in future. Sensitivity of mammography declines drastically in patients with dense breast tissue or small sized tumors [34]. Meanwhile, mammographic density is a high risk factor for breast cancer, especially for small sized breast cancer [35, 36]. In our current research, we found that the sensitivity of mammography is associated with tumor size. When tumor size is more than 2 cm, the sensitivity of mammography achieved to 88.24%, however, when it less than1 cm, the sensitivity declined to 81.82%. It implies that in some breast cancer cases with small sized tumors, the mammography or ultrasound test might neglect them. But the diagnostic sensitivity of methylated gene panel increased modestly along with tumor size decrease. So we hypothesize that breast cancer patients with small sized or early breast tumors (<1 cm) may benefit from the methylated gene panel examination. Prospectively, methylation panel analysis may be a complement with mammography screening, as the sensitivity of which is low in patients with small sized or early breast tumors.

In the analysis of clinical characteristics correlated with the panel methylation positive group, panel methylation positive samples were more frequent in the lower proliferation group (90.29%) and luminal subtypes (84.46%). This association between methylated modification profiles and luminal type breast cancer was similarly referred to many previous investigations [37–39], which suggests that different molecular subtypes of breast cancer would be caused by distinct genetic and epigenetic mechanisms [40]. Moreover, the low proliferation index might also reflected early status of breast cancer.

In summary, the six-marker panel with HOXD13, SFN, RASSF1a, P16, PCDHGB7, and hMLH1 exhibits significantly aberrant methylation in serum cfDNA from breast cancer patients compared with both age-matched healthy women and women with a benign breast disease. However the sensitivity and specificity were not very satisfied to distinguish breast cancers from controls. So our next goal is to develop more useful biomarkers for early breast cancer detection with a higher sensitivity and specificity comparable to the six-gene promoter methylation panel. In addition, other approaches, such as investigations of copy-number variations (CNVs), loss of heterozygosity (LOH), and point mutations in cfDNA, could be also combined to improve the sensitivity and specificity of the panel. Altogether, these approaches could allow for the establishment a biomarker panel offering sensitivity and specificity comparable to that of mammographic examination. Of importance, such a blood-borne screening test would be more convenient for the patient and less expensive for the health-care system.

## MATERIALS AND METHODS

### Study population and clinical characteristics

This study was approved by the Human Research Ethics Committee and Clinical Trials Committee of Harbin Medical University in accordance with the Helsinki Declaration. Written informed clinical research consents were obtained for all patients. All 504 serum samples, except for healthy serum samples, were acquired at the Affiliated Tumor Hospital of Harbin Medical University, Harbin, China from 2009 to 2012, including various sera from 236 benign breast disease patients, including fibroadenoma, desmoid tumors, benign phyllodes tumors, mastopathy, papilloma, duct ectasia and harmatoma. The 245 healthy serum samples were acquired from the Affiliated Tumor Prevention and Treatment Institution of Harbin Medical University, Harbin, China from 2009 to 2012. All 3 sample groups (cancer, benign diseases and healthy controls) were selected from women in the age range 40~60 to rule out the effects of age on DNA methylation [29]. All the breast cancer patients in the study had simultaneously been examined by the mammography and ultrasound before surgery in the hospital. The breast imaging reporting and data system (BI-RADS) diagnosis of mammography and ultrasound followed the standard of the NCCN Breast Cancer Guideline (2015 version).

Immunohistochemistry (IHC) staining for ER, PR and Her-2 were performed as a routine examination in breast cancer clinics. To qualify as Her-2 positive for this study, a case had to demonstrate either a 3+ (strong positive) IHC score or a Her-2 fluorescence in situ hybridization amplification ratio of greater than 2.2. For p53 and ki-67 IHC, only nuclear labeling was scored. For p53, we regarded labeling of >30% of nuclei to be aberrant overexpression (which correlates well but not perfectly with p53 mutation) [41]. The ki-67 cut-off point of 13% was used to designate a tumor as high proliferation subtype [42].

According to the IHC characteristics, cases were categorized into one of 4 categories based upon accepted and previously validated IHC surrogate profiles. Luminal-A tumors were immunoreactive for ER and/or PR, negative for her-2 or low proliferation. ER+ and/or PR+, and either her-2+ and/or high proliferation were considered luminal-B tumors. The subtype of Her-2 defined as ER- and PR-, her-2+. On the basis of published criteria, all the basal-like cases were approximated as triple negative phenotype (ER-/PR-/her-2-). Therefore, we used TNBC instead.

### Collection and processing of samples and DNA preparation

At the enrollment visit, approximately 5 mL of peripheral blood was drawn into a blood collection tube before physical examination or surgery, and all were transferred to the study laboratory within 2 hours of collection for processing. Circulating free DNA (cfDNA) was obtained from 1 mL of serum by using the QIAamp Circulating Nucleic Acid Kit (50) (Qiagen, Hilden, Germany) following the manufacturer's protocols.

### Bisulfite treatment and MethyLight

Bisulfite conversion of DNA was performed using the EZ DNA Methylation kit (Zymo Research, Orange, CA, USA) following the manufacturer's protocols. A detailed list of the nucleotide sequences for MethyLight primers and probes in the promoter or 5′ end region for all analyzed loci is provided in Supplementary Table S2. All probes for the target methylation sites are not detectable in white blood cells (WBC).

TaqMan MGB (Applied Biosystems, Foster City, CA, USA) PCR with primers specific for the bisulfite-converted methylated sequence for a particular locus and with *globin* reference primers were performed separately. TaqMan MGB probes provided a significant improvement in assay specificity, and due to their smaller size, allowed for a more flexible assay design. All analyzed samples were within the different assays' range of sensitivity and reproducibility based on amplification of the internal reference standard (cycle threshold (CT) value for *globin* of 35 or less). The gene of interest was called methylated if the CT of at least two of three PCR replicates for each specimen had a value of less than 40 cycles [43–45]. Genes of interest were considered unmethylated if its CT was not measurable or was ≥ 40. The ratio between the value of the gene of interest and *globin* obtained by TaqMan analysis was used as a measure to represent the relative level of methylation in that particular sample (2^−Δ^^CT^) [43]. The ΔCt was calculated as (CT _Target gene_ – CT _Reference_). The amplification efficiency of the test genes and of the reference gene, *globin*, was examined using serial dilutions of DNA with a 100-fold range and gene-specific primers for each gene and *globin*. All amplification efficiencies were similar (*data not shown*).

Fluorogenic PCRs were carried out in a reaction volume of 20 μL consisting of 600 nmol/L of each primer; 200 nmol/L probe; 0.75 units of platinum Taq polymerase (Invitrogen, Carlsbad, CA); 200 μmol/L each of dATP, dCTP, dGTP, and dTTP; 16.6 mmol/L ammonium sulfate; 67 mmol/L Trizma; 6.7 mmol/L MgCl2; 10 mmol/L mercaptoethanol; and 0.1% DMSO. Two microliters of treated DNA solution was used in each MethyLight reaction. Amplifications were carried out in 96-well plates in a 7500 Sequence detector (Perkin-Elmer Applied Biosystems, Foster City, CA). Each plate consisted of samples and multiple water blanks, as well as positive and negative controls.

### Statistical methods

The methylation frequency comparison was evaluated by Chi-square test. The diagnosis consistency was calculated by Kappa conformance test. Pathological characteristic data comparison used the Student t test, Chi-square test, Fisher exact test, and Mann-Whitney U test. All tests were performed by SPSS 17.0, GraphPad Prism 5.0 (GraphPad Software Inc., La Jolla, CA, USA) and ROC curve analysis was calculated by the RandomForest of R Programming Language (3.2.0). *P* values of <0.05 were considered significant.

For the ROC curve analysis, we divided two main groups, *Cancer vs Benign* and *Cancer vs Health* and two sets for gene promoter methylation analysis were built randomly through the R RandomForest Package, which called the training data set and the test data set. The training data set included 128 breast cancer cases, 114 benign breast disease cases and 112 healthy women cases; the test data set had 125 breast cancer cases, 104 benign breast disease cases and 104 healthy cases. The rest of the samples were abandoned because *globin* was not detected or to eliminate any differences between the training and test data sets with respect to any of the measured demographic or clinicopathologic characteristics, such as age, tumor stage, tumor size, *etc*.

Optimum cutoff values with respect to prediction of case-control status were built in the training data set, and then validated in the test data set and the AUC value was yielded. The final AUC was the mean value through 100 duplicated tests above. The sensitivity of MethyLight-based detection of hypermethylation in serum was calculated as the number of positive tests among the cancer cases divided by the number of total cancer cases. The specificity was calculated as the number of negative tests among the controls divided by the total number of controls.

## SUPPLEMENTARY TABLES


