# Supplementary material for: Detection of aberrant methylation of a six-gene panel in serum DNA for diagnosis of breast cancer
Source: Oncotarget. 2016 Feb 23;7(14):18485–94. doi: 10.18632/oncotarget.7608 (PMC4951303; doi:10.18632/oncotarget.7608)
Supplement: Supplementary file 1 [file oncotarget-07-18485-s001.pdf]

SUPPLEMENTARY TABLES

Supplementary Table S1: The summary of all the target genes

Gene	Gene ID	Full Name	Summary
SFN	2810	stratifin	Diseases associated with SFN include limbal stem cell deficiency and bladder squamous cell carcinoma. Among its related pathways are Regulation of Wnt-mediated beta catenin signaling, P53 signaling pathway and Glucocorticoid receptor regulatory network.
P16	1029	also named CDKN2A (Cyclin-Dependent Kinase Inhibitor 2A)	P16 is a Protein Coding gene. Diseases associated with P16 include melanoma, cutaneous malignant and neural system tumor syndrome. Among its related pathways are Glioma and Pathways in cancer. GO annotations related to this gene include *protein kinase binding* and *p53 binding*.
hMLH1	4292	human MutL Homolog 1	hMLH1 is a Protein Coding gene. Diseases associated with hMLH1 include colorectal cancer, hereditary nonpolyposis and lynch syndrome. Among its related pathways are Pathways in cancer and Cell Cycle / Checkpoint Control. GO annotations related to this gene include *ATPase activity* and *MutSalpha complex binding*.
HOXD13	3239	Homeobox D13	HOXD13 is a Protein Coding gene. Diseases associated with HOXD13 include brachydactyly and syndactyly. GO annotations related to this gene include *sequence-specific DNA binding transcription factor activity* and *chromatin binding*.
PCDHGB7	56099	Protocadherin Gamma Subfamily B, 7	PCDHGB7 is a Protein Coding gene. It is a member of Cluster Protocadherins. GO annotations related to this gene include *calcium ion binding* and *neural system disorder.*
RASSF1a	11186	Ras Association (RalGDS/AF-6) Domain Family Member 1	RASSF1 is a Protein Coding gene. Diseases associated with RASSF1 include intracranial primitive neuroectodermal tumor and monophasic synovial sarcoma. Among its related pathways are Ras signaling pathway and Pathways in cancer. GO annotations related to this gene include *identical protein binding*and *protein N-terminus binding*.

Supplementary Table S2: Nucleotide sequences for methylight primers and probes of all the candidate genes and methylight reaction temperature

Gene	*Forward-Primer*	*Reverse-Primer*	*Production Size*	*Probe*	*Methylation Condition*	
**SFN**	TATGAAAGGCG TCGTGGAGA	GATACTCACGC ACCTCGAACC	179	**AACGTGGTGGGCGGT**	95°c, 3 min	1 cycle
					95°c, 10 secs<br>60°c, 30 secs	40 cycles
**Globin**	AGGTAGAAAAGGA GAATGAAGATAAA	CTTTCCACTCTTT TCTCATTCTCTC	143	**AGGAGGATAAGGAA GAGGGGAAATAGG**	95°c, 3 min	1 cycle
					95°c, 10 secs<br>60°c, 30 secs	40 cycles
**hMLH1**	TGAGGYGGYG ATAGATTAGG	TCCCCTTACRAC CTTTCTAACR	187	**AACGTTGGG TTTATT CGG**	95°c, 3 min	1 cycle
					95°c, 10 secs<br>60°c, 30 secs	40 cycles
**HOXD13**	AGGYGGGAGGT TTATAGAGGG	AACTCCCRAC RCRACTCATA	145	**AGGCGCGTCGCGTT**	95°c, 3 min	1 cycle
					95°c, 10 secs<br>60°c, 30 secs	40 cycles
**RASSF1a**	TGAAGGTTRGAGATT TTTTGTTTTATT	GCAAAACACC AyGyGAAAATA	158	**AGTAAATCGGA TTAGG AGGG**	95°c, 3 min	1 cycle
					95°c, 10 secs<br>60°c, 30 secs	40 cycles
**PCDHGB7**	CGTTATTCGATT TCGGAGGAG	TTCACAAATAA ATCCCCGCTCT	161	**TGGTTAAGGG TTCG GTGGT**	95°c, 3 min	1 cycle
					95°c, 10 secs<br>60°c, 30 secs	40 cycles
**P16**	GGGGTRGGGT AGAGGAGGT	AATCGACCTCC GACCGTAACT	83	**TGTTGTTGG AGGC GGG**	95°c, 3 min	1 cycle
					95°c, 10 secs<br>60°c, 30 secs	40 cycles
